# The Role of the Oral Microbiome in the Development of Diseases

**DOI:** 10.3390/ijms24065231

**Published:** 2023-03-09

**Authors:** Małgorzata Kozak, Andrzej Pawlik

**Affiliations:** 1 Department of Dental Prosthetics, Pomeranian Medical University, Powstańców Wlkp 72, 70-111 Szczecin, Poland; gosia-ko@o2.pl; 2 Department of Physiology, Pomeranian Medical University, Powstańców Wlkp 72, 70-111 Szczecin, Poland

**Keywords:** periodontal diseases, oral microbiome, oral health, oral diseases, systemic diseases

## Abstract

Periodontal disease (PD) is a complex and infectious illness that begins with a disruption of bacterial homeostasis. This disease induces a host inflammatory response, leading to damage of the soft and connective tooth-supporting tissues. Moreover, in advanced cases, it can contribute to tooth loss. The aetiological factors of PDs have been widely researched, but the pathogenesis of PD has still not been totally clarified. There are a number of factors that have an effect on the aetiology and pathogenesis of PD. It is purported that microbiological, genetic susceptibility and lifestyle can determine the development and severity of the disease. The human body’s defence response to the accumulation of plaque and its enzymes is known to be a major factor for PD. The oral cavity is colonised by a characteristic and complex microbiota that grows as diverse biofilms on all mucosal and dental surfaces. The aim of this review was to provide the latest updates in the literature regarding still-existing problems with PD and to highlight the role of the oral microbiome in periodontal health and disease. Better awareness and knowledge of the causes of dysbiosis, environmental risk factors and periodontal therapy can reduce the growing worldwide prevalence of PDs. The promotion of good oral hygiene, limiting smoking, alcohol consumption and exposure to stress and comprehensive treatment to decrease the pathogenicity of oral biofilm can help reduce PD as well as other diseases. Evidence linking disorders of the oral microbiome to various systemic diseases has increased the understanding of the importance of the oral microbiome in regulating many processes in the human body and, thus, its impact on the development of many diseases.

## 1. Introduction

Periodontitis is one of the most common infectious diseases [1], which affects 10–50% of the global population, depending on its severity [2]. Gingivitis is a mild and reversible form of periodontal disease (PD), and if not treated properly, this can progress to periodontitis [3]. PD is a complex and infectious illness that begins with the disruption of bacterial homeostasis. This disease induces a host inflammatory response, leading to damage to the soft and connective tooth-supporting tissues [4,5,6]. Moreover, in advanced cases, it can contribute to tooth loss [7]. The aetiological factors of PDs have been widely researched, but the pathogenesis of PD has still not been totally clarified. There are a number of factors that have an effect on the aetiology and pathogenesis of PD. It is purported that microbiological, genetic susceptibility and lifestyle can determine the development and severity of the disease [8,9]. The human body’s defence response to the accumulation of plaque and its enzymes is known to be a major factor for PD [10]. Microbial plaque is a biofilm that forms on the teeth and gingiva and is one of the crucial causes of PD [11]. There are other individual risk factors contributing to PD such as obesity, poor oral hygiene, stress, a diet low in vitamins C and D and tobacco use [6,10,12,13,14]. The interplay between cigarette smoking and PD has been analysed in numerous studies, suggesting that smoking is a significant environmental risk factor for PD. One component of cigarette smoke, nicotine, can be related to changing clinical aspects and development [6,13]. Additionally, many studies noticed that PD has an impact on the progression of various systemic diseases such as osteoporosis, atherosclerosis, diabetes, cardiovascular diseases and ischemic cardiomyopathy, all of which may aggravate the disease [15,16]. Moreover, other studies revealed the opposite situation, showing that systemic disease can exacerbate PD. In addition, taking medications such as steroids, anti-epilepsy drugs and cancer therapy drugs can also increase PD. Susceptibility to this disorder is connected to the triggering of host antibacterial defence mechanisms [17]. Numerous studies have indicated the association between genetic factors with PD. Cytokines and their genetic polymorphisms influence susceptibility to this disease and its severity. Nevertheless, Nibali et al. presented interesting results of their work, with the inheritance of periodontitis being assessed as OR 0.38 (95% CI, 0.34–0.43) in twin studies and OR 0.15 (95% CI, 0.06–0.24) in other family research [18]. It was observed that changes in the oral microbiome composition naturally increase with age [19], which may be related to the greater susceptibility of the elderly to chronic periodontitis [20]. Periodontal disorders intensify problems with chewing, the function of speech and aesthetics, which definitely worsen the quality of life [19].

A new model of the pathogenesis of periodontal disorders assumes that the disease involves a more diverse microflora associated with periodontitis than previously thought. The disease is caused by the synergy of multiple microbes and dysbiosis, which disrupt the ecologically balanced biofilm associated with periodontal homeostasis and are not the result of individual pathogens [21]. Under healthy conditions, the oral microbiome exhibits a well-balanced, dynamic ecosystem [22]. Dysbiosis of the oral microbiome means an imbalance in relative abundance or an impact on microbial species, which contribute to the disease development of susceptible patients [23].

## 2. Microbiome in the Oral Health

The term “microbiome” was created by Joshua Lederberg and refers to the community of symbiotic, commensal and pathogenic microorganisms [24]. The composition and interactions of any microbiome contribute to overall health, being a key factor in oral health [25]. The oral cavity is colonised by a characteristic and complex microbiota that grows as diverse biofilms on all mucosal and dental surfaces [26]. There are more than 700 species of bacteria, fungi, viruses, archaeobacteria and protozoa in the oral cavity [20]. Bacteria are the most well-researched microorganisms in the oral cavity [27], but only 57% of bacterial species in the oral cavity have been officially named [28]. In health, oral microflora mainly consists of facultative anaerobic Gram-positive bacteria [29]. The oral fungal microbiome (mycobiome) is a significant component of the oral microbiome. The *Candida* genus is present in about 25–75% of healthy individuals as a commensal organism [30]. *Candida albicans* is one of the most crucial, prevalent fungal species. Under certain, favourable conditions, *Candida* species, as opportunistic pathogens, can cause infections of the oral mucosa [31]. The oral microbiota usually live in harmony with the host and provide important benefits that contribute to overall health. The microorganisms in oral biofilms do not exist as single cells but live in close proximity to one another [27]. The microbial interactions can be synergistic or antagonistic [32,33]. Moreover, the oral environment has an impact on the composition of the microbiome. If some changes in local conditions occur, they can influence interactions between microorganisms in the mouth and increase the risk of PD. The composition of the oral microbiome has been widely explored by using metagenomics and metatranscriptomics [2]. Using these methods, Belstrøm et al. observed the transcriptional activity of prevalent *Streptococcus* species under healthy conditions and periodontitis. The researchers discovered that the transcriptional activity of *Streptococcus* species was higher in health and reduced in PD [34]. Of particular note, *Streptococcus* species are Gram-positive, aerobic to facultatively anaerobic bacteria that are part of the normal flora of the oral cavity. In health, new species of *Streptococcus dentisani* and Streptococcus salivarius, which have potential probiotic features, are associated with the treatment of miscellaneous oral diseases such as periodontal disorders, among others [35,36,37].

Recently, there has been growing interest in the use of probiotics to treat PDs. Probiotics are defined as “living microorganisms that can have a beneficial effect on the host when taken in sufficient doses” [38]. Their function is to regulate host immune function, restore balance and maintain homeostasis in the mouth [3]. Good results of probiotics in improving oral health have been noticed not only in periodontal disorders [39] but also in dental caries [40], *Candida* infection [41] and halitosis [42]. The genus *Lactobacillus* is well known as a health-promoting probiotic in periodontal therapy. *Bifidobacterium*, *Streptococcus* and *Weissella* are also known probiotics, which play a positive role in oral care. Other species such as *Bacillus subtilis* and *Saccharomyces cerevisiae* also have a good impact on the oral cavity [3]. In addition, some strains of bacteria isolated from the oral cavity have been produced commercially as probiotics, including *Lactobacillus reuteri*, *Lactobacillus brevis* and *Streptococcus salivarius* [25,43]. Kawai et al. [44] suggested that *Limosilactobacillus (Lactobacillus) fermentum* ALAL020 may be a future probiotic candidate. This bacterium produces a cyclic dipeptide with antibacterial activity against *Porphyromonas gingivalis* and *Prevotella intermedia* [44]. Currently, the beneficial effects of synbiotics on health are of great interest to scientific research. Synbiotics are defined as “a mixture comprising live microorganisms and substrate(s) selectively utilized by host microorganisms that confers a health benefit on the host” [43]. It has been observed that administering a synbiotic in combination with probiotics can help prevent and treat certain metabolic disorders. However, there is little evidence for this [45]. Duraisamy et al. noticed that synbiotics can diminish *Streptococcus mutans* levels in children’s saliva but are less effective compared to probiotics [46].

## 3. Oral Microbiome in Periodontal Diseases

The triad of oral anaerobic bacteria, the so-called “red complex” (*Porphyromonas gingivalis*, *Treponema denticola and Tannerella forsythia*), have historically been considered as the basic infectious organisms associated with periodontitis [27]. However, this has been identified in culture-based studies, and many of the wide variety of bacteria present in samples were overlooked [47]. Nevertheless, only a few bacteria, namely *P. gingivalis*, *Aggregatibacter actinomycetemcomitans*, *Tannerella forsythia*, *Prevotella intermedia* and *Fusobacterium nucleatum*, were confirmed to initiate and progress PDs [48]. In addition, *Candida albicans* is one of the most crucial fungal residents of the oral microbiome. This commensal coloniser protects *P. gingivalis* from being recognised by the host’s immune cells and can contribute to bacterial infections of the gums [29]. Contemporary periodontology concentrates not only on the pathogenicity of dental plaque but also on the interplay between oral microorganisms and the host [3]. Microflora perturbances then lead to gingivitis and, eventually, periodontitis. Factors such as the availability of oxygen, nutrients and changes in pH may contribute to disorders in homeostasis in the oral cavity as well as systemic diseases [47,49]. Modification in the oral microbiome can lead to the expansion of microorganisms and provides excellent conditions for the growth of opportunistic microbes [10]. In addition, perturbations in the periodontal microbiota are associated with an alteration from a symbiotic to dysbiotic microbial community. The symbiotic structure includes facultative bacteria such as *Actinomyces* and *Streptococcus*, which then shifts to mainly anaerobic types (such as the *phyla Firmicutes*, *Proteobacteria*, *Spirochaetes*, *Bacteroidetes* and *Synergistetes*) [48]. The transition of microbial composition precedes the clinical symptoms of PD [50]. It is widely known that factors such as residing microorganisms, age, general health, lifestyle, and nutritional status have an impact on oral health [50].

In approximately 90% of cases of PD, halitosis (malodour) can be noticed in the mouth [51]. Halitosis is mainly caused by *P. gingivalis*, *T. denticola*, *Fusobacterium* and *T. forsythia*, the same species that have been associated with PD. The process of bacterial degradation of sulphur-containing amino acids into volatile gases leads to oral malodour. Halitosis is caused by the biodegradation of sulphur-containing amino acids and the production of volatile sulphur compounds [52]. Moreover, poor oral hygiene, bacterial coating of the tongue and periodontal disorders such as gingivitis, periodontitis and caries can contribute to halitosis [53]. Malodour can also be affected by dietary habits, such as smoking, alcohol consumption, obesity, diabetes, stress and advanced age [54,55,56]. In addition, risk factors such as age and tooth decay can also lead to malodour in children [57], but it is difficult to distinguish between oral bacteria that cause odour in adolescents. Three types of bacteria including *Fusobacterium*, *Veillonella* and *Prevotella* are dominant in children with halitosis [56]. A noteworthy finding observed by Wu et al. is that the oral microbiome was altered, and more abundant species were present among obese people suffering from malodour compared to healthy persons [58]. Tobacco use has a similar effect and may also alter the diversity of the oral microflora. Yitzhaki et al. observed the relationships between patients wearing dentures and halitosis [59]. The researcher noticed higher bacterial diversity in the oral microflora among patients with halitosis wearing dentures, observing meaningful differences from the control group. There were bacterial taxa, including 117 species, 29 genera (mainly *Leptotrichia*, *Megasphaera*, *Atopobium* and others) and 9 phyla (*Fusobacteria*, *Firmicutes*) detected [59]. An important research finding was that *Candida* species accounted for the largest percentage of microbes among smokers with halitosis [56]. Zhang et al. suggested that halitosis can be detected long before clinical symptoms appear as a result of changes in the microbiome of the tongue coating. Nevertheless, alterations in the tongue coating microbiome can be used as biomarkers of an early stage of halitosis and can help find better strategies for the diagnosis, prognosis and treatment [60]. Although periodontitis is associated with a variety of microorganisms, *Fusobacterium nucleatum* is a known, predominant periodontal pathogen that can influence other bacteria and form an inflammatory microenvironment. In addition, *F. nucleatum* may modulate and enhance the invasive potential of *P. gingivalis* [61]. Thus, it can promote and accelerate the development of periodontitis. Moreover, *Fusobacterium nucleatum* can cause local halitosis and pulp infections and may systemically promote the development or progression of oral cancer and other extraoral diseases [62]. Kang et al. [63] explored and identified three types of *Weissella cibaria* from human saliva, which produce hydrogen peroxides. These isolates can inhibit volatile sulphur compounds formed by *F. nucleatum*. In addition, *W. cibaria* can block the production of interleukin-6 and interleukin-8 by oral epithelial cells caused by *F. nucleatum*. Suzuki et al. [64] showed that *Lactobacillus saliva WB21* buccal tablets can specifically reduce the amount of *F. nucleatum* in patients with halitosis. This means that the use of *W. cibaria* and *Lactobacillus saliva WB21* as probiotics can be a beneficial method of combating bad breath and controlling PD [62,64].

Subgingival communities of microbes, including fungi, archaea and viruses, can lead to periodontitis-related dysbiosis [8]. The microbial communities are responsible for the pathological processes that have an impact on the periodontium. Although alterations in the composition and function of subgingival bacteria have been extensively explored, how the succession of microorganisms and the transformation of health into disease proceeds is still not fully understood. Periodontal health is frequently defined as opposite to PD, in the absence of any clinical symptoms of disorder [65]. The development of the oral microflora is known to involve interactions between the host’s genetics and the host’s immune system, and changes in the composition of the microbiome depend on exposure to environmental factors [10].

During periodontal health, the health-associated species dominate the local microbiome. The development of gingivitis or PD is associated with increased biomass and the appearance of pathogenic species. The further progression of the disease is characterized by a shift in the microbiome balance into PD-associated pathogens. Alterations in composition and species diversity may lead to the identification of potential biomarkers in the diagnosis of PDs [55]. In addition, biomarkers in saliva may reflect various conditions in the oral cavity connected with periodontitis. Saliva easily collects and can show the condition of the entire mouth. For example, nitric oxide (NO) is known as a biological marker, which is related to the aetiopathogenesis of oral diseases [66]. Reher et al. observed increased levels of NO in patients with periodontal disorders in comparison to healthy people [67]. Furthermore, it was noticed that the level of NO was correlated with periodontitis severity. The researcher also suggested that the levels of NO were linked with the deterioration of periodontal parameters such as probing depth and were the result of an inflammatory response induced by bacteria.

## 4. Oral Microbiome in Systemic Diseases

Under different conditions, bacterial flora has the ability to change the balance between health and sickness, both locally and systemically. Microorganisms present in the mouth interact with themselves and with the host in illness and health [68]. The oral cavity is the entry point and direct way to the lungs and the digestive system, so the microbiomes of these structures are interconnected across the human body. That explains why the oral microbiome is associated with many systemic diseases [47]. Homeostasis disorders due to food habits and poor oral hygiene not only lead to oral diseases such as caries or periodontal problems but also to other systemic diseases and cancers. The direct route and good access of the oral microbiome to the respiratory system explain the link between oral disorders and lung diseases, such as respiratory tract infection, bacterial pneumonia, chronic obstructive pulmonary disease (COPD) and cystic fibrosis [69].

Previous studies have shown that disturbances in the oral microbiome can lead to abnormalities in the airway microbiome, which can cause an abnormal local immune response and chronic inflammation in the airways and the onset of chronic obstructive pulmonary disease (COPD) [70]. The inflammatory process caused by bacterial infection leads to impaired lung defence mechanisms, contributing to progressive lung damage and the loss of lung function, which is characteristic of COPD [71]. In COPD, there is a higher incidence of bacterial colonization by potential respiratory pathogens, such as *Pseudomonas aeruginosa* and bacteria of the genus *Actinomyces* [72].

*Pseudomonas aeruginosa* is an important pathogen in cystic fibrosis patients. Whiley et al. have found that in vitro *Streptococcus* species could modulate the production of virulence factors (elastase and pyocyanin) by *P. aeruginosa* [73]. It has also been shown that the pathogenicity of *P. aeruginosa* can be inhibited by *S. oralis* through the production of hydrogen peroxide (H_2_O_2_) [74].

Furthermore, Haran et al. presented intriguing findings that the dysbiosis of the oral microbiome may affect the duration of COVID-19 symptoms [75]. *Prevotella* species have been found in abundance in COVID-19 patients. Furthermore, these species are considered to produce proteins that can contribute to SARS-CoV-2 infection and may aggravate COVID-19 [76]. *Veillonella* strains are also capable of eliciting inflammatory responses. This genus induces IL-6 (Interleukin-6) [77], whereas *Prevotella* species activate TLR-2 and the expression of IL-23 and IL-1 [78].

**Figure 1:**
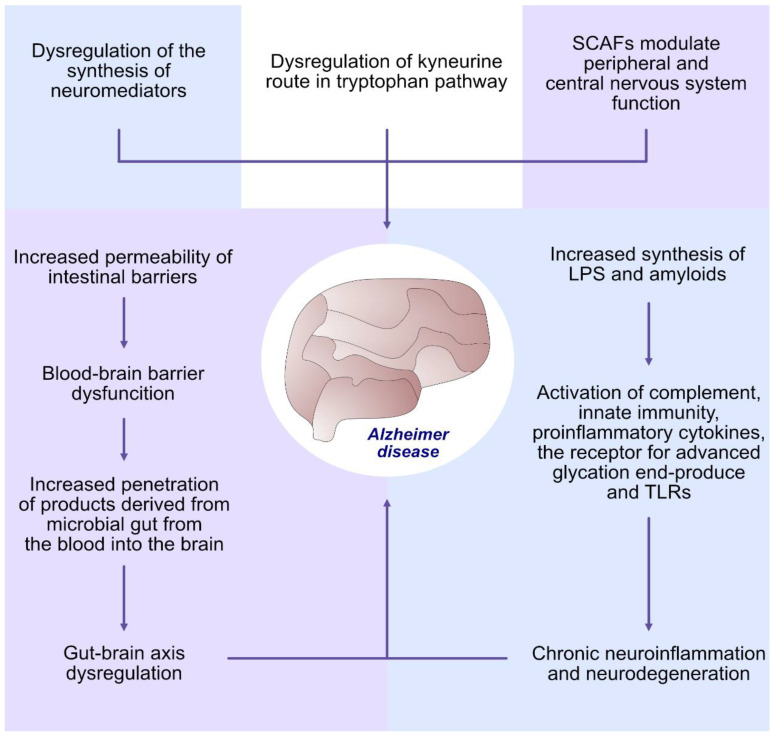
The involvement of oral microbiome dysbiosis in the development of Alzheimer’s disease. SCFAs—short-chain fatty acids, TLRs—toll-like receptors, LPS—lipopolysaccharide.

Studies in recent years have shown that bacteria produce a number of compounds that cause the development of systemic inflammatory responses that impair the blood–brain barrier (BBB), exacerbating neuroinflammation and ultimately neurodegeneration [79,80,81]. The microbiota produces a number of neuromediators such as serotonin, kynurenine, melatonin, GABA (gamma-aminobutyric acid), tryptophan, catecholamines, histamine and acetylcholine [82,83]. Abnormalities in the serotonin and kynurenine pathway of tryptophan metabolism have been detected in patients with neurodegenerative diseases including Alzheimer’s disease (AD) [84]. Dysregulation of the kynurenine pathway of tryptophan metabolism may be one of the main factors contributing to AD development [85,86]. Other metabolites produced by the microbiota that can affect brain function and blood–brain barrier permeability are short-chain fatty acids (SCFAs): acetate, butyrate and propionate [87,88]. SCFAs can affect transmission processes in the central nervous system and thus regulate cognitive function. LPS and amyloids secreted by bacteria are involved in the process of neurodegeneration [89,90]. Many bacterial species produce extracellular amyloid fibres to form a biofilm [91]. Bacterial and brain amyloids are biologically similar in structure, composition and physicochemical characteristics [90]. Previous studies have shown that secretory products from the microbiome exert strong pro-inflammatory effects by activating complement and other components of the immune response, leading to an increased synthesis of pro-inflammatory cytokines and the development of neuroinflammation in the brain [92,93]. This intensifies amyloid aggregation and inflammatory responses. Both bacterial amyloid proteins and LPS are potent activators of the chronic inflammation in the cerebral rim observed in AD patients [94]. Dominy et al. [95] observed a correlation between *P. gingivalis* and the progression of Alzheimer’s disease. The presence of this bacterium was noticed in the brain of AD patients. During studies in mice, it was also discovered that the infection of *P*. *gingivalis* led to brain colonisation and the growth of components of amyloid plaques. Nevertheless, *P. gingivalis* produces neurotoxic gingipain proteases, which inhibit tau function (a hallmark of AD). This means that gingipain inhibitors could be used to treat neurodegeneration in AD. Furthermore, oral species of the phylum *Spirochaetes* also form amyloid plaques and play a role in the progression of dementia in AD. In addition, these organisms avoid host defence and create more atypical, resistant forms and biofilms, which contribute to the maintenance of chronic infection and higher resistance to treatment [96] (Figure 1, Table 1 and Table 2).

**Figure 2:**
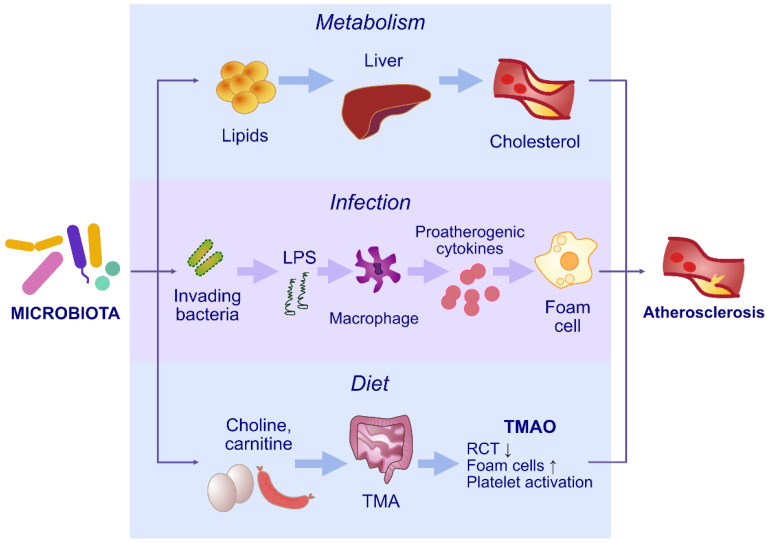
The involvement of oral microbiome dysbiosis in the development of atherosclerosis. LPS—lipopolysaccharide, TMAO—trimethylamine N-oxide, TMA—trimethylamine, RCT—reverse cholesterol transport.

The potential impact of periodontal infection on cardiovascular diseases has been the subject of much research [97,98]. Teles et al. undertook the challenge of explaining possible associations with an increased risk of cardiovascular diseases and periodontal disorders. However, this process is still poorly clarified [99]. Much is known about individual pathogens connected with periodontitis, rather than the mechanisms describing the association between PDs and cardiovascular disease. It has been shown that a number of compounds produced by the microbiome can enhance the development of atherosclerotic lesions in the vessels. Such compounds include trimethylamine (TMA), which is oxidized by monooxygenase to TMAO (Trimethylamine N-oxide) [100]. TMAO enhances foam cell formation and increases VCAM-1 (Vascular cell adhesion molecule-1) expression, which enhances monocyte adhesion to the endothelium [101]. There is also the activation of the protein kinase C (PKC) and nuclear factor-κB (NF-κB) pathway, which disrupts endothelial cell function and results in the development of atherosclerotic lesions [102,103]. Proatherogenic effects are also exhibited by SCFAs, which affect the processes of chemotaxis and phagocytosis, induce the formation of reactive oxygen species and activate monocytes and macrophages [104,105]. Another component that exacerbates atherogenesis may be LPS present on the cell membrane of bacteria. LPS increases the expression of chemokines and adhesion molecules, enhances the formation of foam cells and increases the adhesion of monocytes to endothelial cells [106]. In addition, LPS can also bind to toll-like receptor 4 (TLR4) on the surface of immunocompetent cells and induce the secretion of pro-inflammatory cytokines (TNF, IL-6) [105]. This leads to the development of inflammation in the vessels and the formation of atherosclerotic plaque, affecting its stability (Figure 2, Table 2).

**Figure 3:**
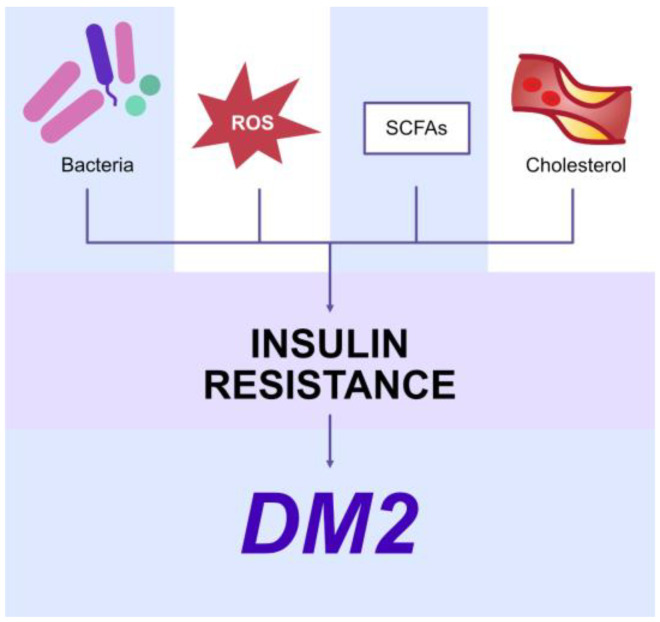
The involvement of oral microbiome dysbiosis in the development of diabetes mellitus. ROS—reactive oxygen species, SCFAs—short-chain fatty acids, DM2—diabetes mellitus type 2.

In addition, the microbiome has been shown to affect lipid and carbohydrate metabolism. It has been shown that SCFAs can modulate pancreatic β-cell function and insulin production, contributing to the development of diabetes [106]. SCFAs stimulate parasympathetic nervous system functions to increase food intake [107]. Products of the microbiome can also cause disturbances in bile acid circulation [108]. Microbiome dysbiosis may also stimulate chronic systemic inflammation and enhance oxidative stress, leading to insulin resistance and the development of diabetes [109] (Figure 3, Table 2).

Xiao et al. presented the interplay between the oral microbiome, diabetes and PDs [110]. The researcher observed that the pro-inflammatory cytokine IL-17 is associated with periodontitis and that its inhibition has an impact on the pathogenicity of the diabetic microbiome. Moreover, diabetes exacerbates periodontitis, while periodontitis causes a pathogenic alteration in the microbiome. This biome change contributes to the susceptibility and severity of PD. Treatment with anti-IL-17 antibodies may alleviate symptoms.

Recent studies have shown that periodontal disease is correlated with an increased risk of rheumatoid arthritis (RA) in humans and in mouse models of arthritis [111]. Rheumatoid arthritis is a systemic inflammatory disease that leads to joint destruction. The pathogenesis of this disease has been shown to be complex, with both genetic and environmental factors involved in its development. One of the environmental factors involved in the development of RA has been shown to be the microbiome [112]. Studies in recent years have shown a higher incidence of RA in patients with periodontal disease [113,114]. In addition, it has been shown that the severity of periodontal disease correlates with the activity of the disease process in RA patients and that treating the symptoms of periodontal disease results in the alleviation of RA symptoms [115,116]. These data suggest that bacteria associated with the development of periodontal disease may be involved in the pathogenesis of RA.

**Figure 4:**
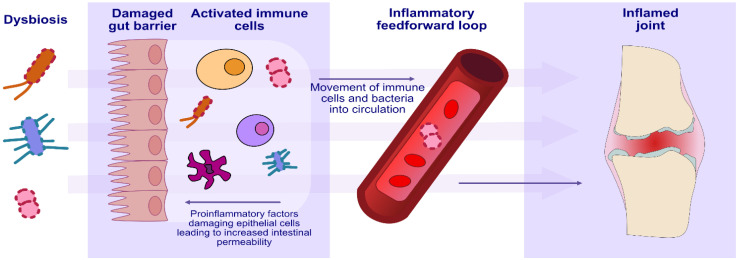
The involvement of oral microbiome dysbiosis in the development of rheumatoid arthritis.

One of the main bacteria involved in the development of periodontal disease is *Porphyromonas gingivalis.* It has been shown that this bacterium can induce the formation of anti-CCP antibodies in RA patients [117,118,119,120]. In addition, *Porphyromonas gingivalis* can enhance the production of IL-17, a cytokine that plays an important role in the development of inflammation in RA [121]. *Porphyromonas gingivalis* was also found to enhance the formation of Th17 cells involved in the pathogenesis of RA [121]. Another bacterium that is thought to be associated with the pathogenesis of RA is *Aggregatibacter actinomycetemcomitans*. This bacterium has been shown to induce the formation of citrullinated autoantigens, which play a key role in the development of RA [122,123]. Konig et al. showed that leukotoxin-A produced by *A. actinomycetemcomitans* enhances hypercitrullination in neutrophils, an important element in the development of a pathological immune response in RA [124]. In addition, antibodies to *A. actinomycetemcomitans* and leukotoxin-A have been found in RA patients [125]. In conclusion, periodontal bacteria such as *Porphyromonas gingivalis* and *Aggregatibacter actinomycetemcomitans* may be involved in the development of RA by contributing to the production of autoantibodies and the process of autoimmunity. Further research is needed on the involvement of the microbiome in the pathogenesis of RA (Table 1 and Table 2, Figure 4).

**Figure 5:**
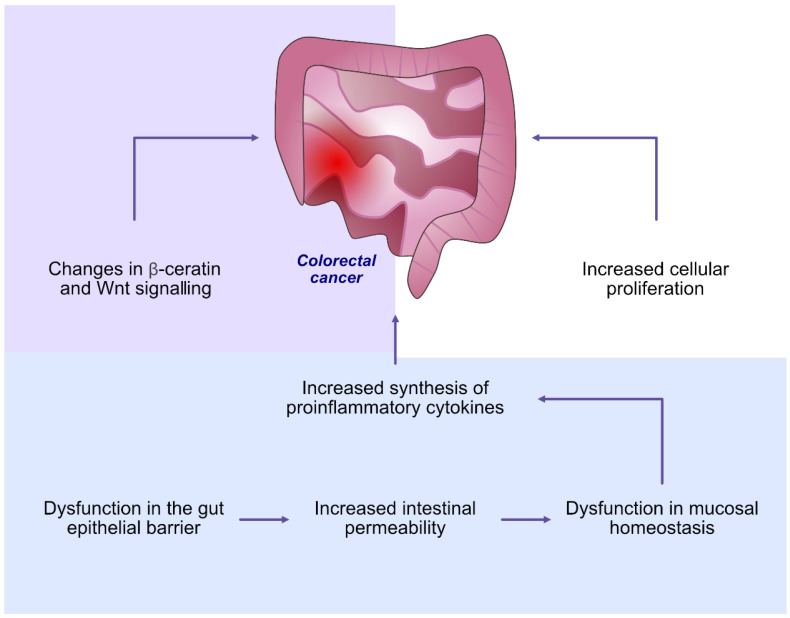
The involvement of oral microbiome dysbiosis in the development of colorectal cancer.

The oral microbiome plays a significant role not only in maintaining oral health but also in maintaining balance throughout the body. Many studies presented findings showing that the dysbiosis of oral microbiota may also contribute to the development of cancers [126,127]. Farrell et al. [128] noticed reduced levels of *Neisseria elongata* and *Streptococcus mitis* in the salivary microbiome in patients with pancreatic cancer compared to healthy individuals, while the levels of *Granulicatella adiacens* were significantly higher in patients with pancreatic cancer. Previous studies have also shown that the dysbiosis of the oral microbiome may be involved in the development of colorectal cancer. The disturbances in the oral microbiome may cause dysfunction in the gut epithelial barrier, increased intestinal permeability, increased synthesis of pro-inflammatory cytokines, increased cellular proliferation, as well as changes in β-catenin and Wnt signalling, leading to enhanced carcinogenesis [129,130] (Table 1 and Table 2, Figure 5).

## 5. Conclusions

Previous studies highlight the role of the oral microbiome in periodontal health and disease. Greater awareness and knowledge of the causes of dysbiosis, environmental risk factors and periodontal therapy can reduce the increasing prevalence of PD worldwide. The promotion of proper oral hygiene, a reduction in smoking, alcohol consumption and exposure to stress and comprehensive treatment to reduce the pathogenicity of the oral biofilm can help reduce the incidence of PD. Recent research on the human microbiome has led to increased interest in the oral microbiome and its impact on the normal course of oral processes and the development of disease states, including many systemic diseases. Evidence linking the relationship between disorders of the oral microbiome and various systemic diseases has increased awareness of the importance of the oral microbiome in regulating numerous processes in the human body and, thus, its impact on the development of many diseases. Full knowledge of the role of the oral microbiome in health and the development of disease processes can contribute to the prevention and treatment of diseases.

## Figures and Tables

**Table 1:** The associations between oral bacteria and diseases.

General Diseases	Bacteria	References
Respiratory tract infection Chronic obstructive pulmonary disease Cystic fibrosis	*Porphyromonas gingivalis * *Aggregatibacter actinomycetemcomitans* *Fusobacterium nucleatum* *Chlamydia pneumoniae*	[69,70,71,72,73]
Alzheimer’s disease	*Prevotella intermedia* *Tannerella forsythia* *Aggregatibacter actinomycetemcomitans* *Porphyromonas gingivalis* *Fusobacterium nucleatum*	[79,80,81,82,83,84,85,86,87,88,89,90,91,92,93]
Cardiovascular diseases (atherosclerosis/coronary diseases)	*Porphyromonas gingivalis* *Treponema denticola* *Aggregatibacter actinomycetemcomitans* *Prevotella intermedia* *Tannerella forsythia*	[97,98,99,100,101,102,103,104,105,106]
Diabetes and insulin resistance	*Porphyromonas gingivalis* *Aggregatibacter actinomycetemcomitans* *Fusobacterium nucleatum*	[107,108,109,110]
Rheumatoid arthritis	*Porphyromonas gingivalis* *Aggregatibacter actinomycetemcomitans*.	[111,112,113,114,115,116,117,118,119,120,121,122,123,124,125]
Pancreatic cancer Colorectal carcinoma	*Neisseria elongata* *Granulicatella adiacens* *Porphyromonas gingivalis* *Fusobacterium nucleatum*	[126,127,128,129,130]

**Table 2:** The involvement of oral microbiome dysbiosis in the development of diseases.

General Diseases	Mechanisms	References
Atherosclerosis and coronary artery disease	LPS induces the expression of chemokines and cell adhesion molecules Increased production of trimethylamine Increased foam cell generation Promoting monocyte adherence by up-regulating the level of vascular cell adhesion molecule-1 (VCAM-1) Activation of the protein kinase C (PKC) and nuclear factor-κB (NF-κB) pathways Damage to endothelial cells Disturbances in mitochondrial repair and myocardial metabolism Disturbances in bile acid circulation Disturbances in cholesterol and lipid metabolism Enhanced synthesis of pro-inflammatory cytokines Inflammatory response in endothelium Promotion of atherosclerotic plaque formation Plaque instability	[97,98,99,100,101,102,103,104,105,106]
Alzheimer’s disease	Dysregulation of the synthesis of neuromediators: serotonin, kynurenine, melatonin, GABA, catecholamines, histamine and acetylcholine Dysregulation of kynurenine route in tryptophan pathway Short-chain fatty acids (SCFAs), (acetate, butyrate and propionate) modulate peripheral and central nervous system function Increased permeability of intestinal barriers Blood–brain barrier dysfunction Increased penetration of products derived from microbial gut from the blood into the brain Gut–brain axis dysregulation Increased synthesis of LPS and amyloids Activation of complement, innate immunity, pro-inflammatory cytokines, the receptor for advanced glycation end-products (RAGE) and TLRs. Chronic neuroinflammation and neurodegeneration	[79,80,81,82,83,84,85,86,87,88,89,90,91,92,93]
Diabetes and insulin resistance	Short-chain fatty acids act on parasympathetic activity to increase food intake Stimulation of TLR-4 by bacterial LPS induces inflammatory response Disturbances in bile acid circulation Disturbances in cholesterol and lipid metabolism Enhanced synthesis of pro-inflammatory cytokines Chronic systemic inflammation Enhanced oxidative stress Insulin resistance	[107,108,109,110]
Rheumatoid arthritis	Induction of anti-CCP antibodies Hypercitrullination in neutrophils Increased production of IL-17 Formation of Th17 cells	[111,112,113,114,115,116,117,118,119,120,121,122,123,124,125]
Colorectal carcinoma	Dysfunction in mucosal homeostasis Dysfunction in the gut epithelial barrier Increased intestinal permeability Increased synthesis of pro-inflammatory cytokines Increased cellular proliferation Changes in β-catenin and Wnt signalling	[126,127,128,129,130]

## Data Availability

Not applicable.
